# Examining hurricane–related social media topics longitudinally and at scale: A transformer-based approach

**DOI:** 10.1371/journal.pone.0316852

**Published:** 2025-01-24

**Authors:** Dhiraj Murthy, Sophia Elisavet Kurz, Tanvi Anand, Sonali Hornick, Nandhini Lakuduva, Jerry Sun

**Affiliations:** 1 Moody College of Communication, Department of Sociology, and School of Information, University of Texas at Austin, Austin, Texas, United States of America; 2 Computational Media Lab, University of Texas at Austin, Austin, Texas, United States of America; University of Zagreb School of Medicine: Sveuciliste u Zagrebu Medicinski fakultet, CROATIA

## Abstract

Instead of turning to emergency phone systems, social media platforms, such as Twitter, have emerged as alternative and sometimes preferred venues for members of the public in the US to communicate during hurricanes and other natural disasters. However, relevant posts are likely to be missed by responders given the volume of content on platforms. Previous work successfully identified relevant posts through machine-learned methods, but depended on human annotators. Our study indicates that a GPU-accelerated version of BERTopic, a transformer-based topic model, can be used without human training to successfully discern topics during multiple hurricanes. We use 1.7 million tweets from four US hurricanes over seven years and categorize identified topics as temporal constructs. Some of the more prominent topics related to disaster relief, user concerns, and weather conditions. Disaster managers can use our model, data, and constructs to be aware of the types of themes social media users are producing and consuming during hurricanes.

## Introduction

Hurricanes are among the most devastating natural disasters. In just several weeks during late 2024, Hurricanes Helene and Milton caused significant damage to the southeastern United States. Hurricane Helene had 140 mph winds and heavy rainfall that caused power outages to over 3.5 million people, major damage to structures, and led to over 225 people dead across several states in the southeastern U.S. [1, 2] In 2017, Hurricane Harvey left 72,000 people in need of assistance in Houston, Texas [3]. Hurricane Harvey highlighted a critical issue: the overwhelming of traditional emergency systems like the 9–1-1 telephone system, which received up to 10 times the normal call volume [4]. This overload led to a significant gap in communication and coordination between emergency services and government agencies, as noted by disaster victims [5].

The slow response times of emergency services in such situations have shifted public behavior. Increasingly, people are using social media, not just as a means of spreading information about disasters, but as a primary communication channel for seeking help [6]. This shift is significant, as social media has been shown to influence volunteer participation during disasters greatly [7]. However, this reliance on social media for crisis communication also presents challenges, particularly in terms of the coordination between volunteers and official emergency response channels.

The promise and challenges of using unconventional data sources for large-scale analysis are not new. The case of “Google Flu Trends,” which attempted to predict flu outbreaks based on search engine data, illustrates the potential risks associated with inconsistent data collection and overreliance on indirect signals. While the project initially showed promise, it encountered significant difficulties due to changes in user behavior and methodological shortcomings [8]. This example underscores the importance of rigorously structured and consistent methodologies when analyzing alternative data sources. Building on this lesson, our study employs a systematic approach to social media data collection and analysis, ensuring that insights are reliable and adaptable to disaster scenarios.

When disasters occur, the immediacy and informality of social media platforms like Twitter become critical for people to share their experiences and seek assistance [9]. Communication through these platforms plays a vital role in disaster planning, response, and recovery [10]. In some cases, victims perceive social media as their only access to help [11]. Monitoring social media provides a unique insight into the different phases of a hurricane from the perspective of those directly affected. This information, particularly when analyzed over time, can bolster resilience in future disasters.

One significant challenge during disasters is the use of social media images and videos. While these can help differentiate between critical and non-critical information [12], real-time classification and the need for high-quality equipment, fast internet connections, and sufficient battery power make it difficult for victims to post useful images. These limitations highlight the need for real-time data access and analysis to understand and better aid disaster victims.

This study leverages Twitter data from four US hurricanes over seven years, aiming to make the following contributions:

1. The application of a GPU-accelerated version of BERTopic, a deep learning topic modeling method, to analyze large-scale hurricane-related social media datasets.
2. The successful derivation of thematic constructs from BERTopic, categorizing these by disaster phases: warning, recovery, and response.
3. Providing a model and data that can be utilized by disaster management organizations to understand the types of themes prevalent in social media communications during hurricanes.

This research demonstrates the value of using social media APIs to filter and analyze data beyond basic hashtags, providing more targeted information for first responders [13]. It hypothesizes that tweets from different hurricane phases have common keywords and phrases, knowledge that can be vital for both volunteers and formal responders in areas like emotional support, material support, and resource allocation.

Technological advancements have made social media a critical tool for both peer-to-peer and one-to-many communication during disasters [14]. Its ubiquity in everyday life enables the mass sharing of both trivial and critical information, especially during natural disasters [15]. This study evaluates the use of machine learning models, particularly BERTopic, to understand and model tweets during natural disasters. Our findings show that BERTopic effectively differentiates tweets into specific disaster phases, providing valuable surveillance trends for stakeholders like first responders and policymakers [16].

### Theoretical contributions

Our study’s methodological novelty lies in its extension of BERTopic to a diverse set of hurricane tweets. We provide a construct of hurricane tweet themes that can be used by policymakers and disaster managers to anticipate some of the content that will be posted during disasters on social media. The advantage of this construct is that all interpretation has been done as a one-step procedure, and any future classifications that are done using our pre-trained model will directly follow this construct. There also remains a continued need to study communication while a hurricane is taking place, rather than solely post-disaster.

### Research questions

Based on the potential of unsupervised methods illustrated by [13], we propose the following research questions:

Research Question 1 (RQ1): Is it possible to identify a distinct set of topics within tweets related to hurricane disaster relief? Can these topics be accurately and automatically detected using advanced machine learning algorithms and statistical methods, thereby eliminating the need for human annotation in the process? This question is rooted in the need for rapid information processing during disasters, recognizing that the speed and accuracy of identifying relevant social media content can significantly impact the efficiency of disaster response efforts [3, 4].

Research Question 2 (RQ2): Can we effectively distinguish between tweets corresponding to different phases of a hurricane, and how can this differentiation contribute to improving disaster response? This question explores the potential of using real-time social media data to provide nuanced insights into various stages of a disaster, thereby aiding first responders and other stakeholders in making informed decisions quickly [5, 6].

These research questions are driven by the critical need to enhance the response capabilities of emergency services. Accurate and timely information is vital during natural disasters, where every moment counts in reaching and assisting victims. Our study aims to assess the feasibility and benefits of leveraging social media data, specifically Twitter, to provide enriched information to first responders. This enrichment could potentially reduce the response time during hurricane events, a factor crucial for the safety and well-being of affected individuals [7, 9].

Furthermore, many response organizations are currently hesitant to integrate social media-derived insights due to limited resources and trust issues, particularly during large-scale events. By examining tweets across multiple hurricanes and their respective phases, our study seeks to demonstrate the practical utility of analyzing these longitudinal data. Such insights could potentially offer new knowledge specific to different disaster stages, aiding in more targeted and effective response strategies [12, 13].

In addressing these questions, we also aim to provide findings that are not only theoretically sound but practically applicable. We endeavor to present results that resonate with and are evaluated by response organizations and policymakers, thereby bridging the gap between academic research and on-ground disaster management practices [15, 16].

## Literature review

### Social media and disasters research

Natural disasters, epidemics, and pandemics change the lives of many people all over the world. Many people globally turn to social media to express their thoughts in times of crisis. The COVID-19 pandemic has been a recent example of this and Twitter data has been used, for example, to study the impact of the pandemic on working professionals [17] sports events [18], and mental health [19]. In addition to being a means of communicating, social media also serves as an informational platform that can promote disaster relief efforts, such as donating funds towards relevant charities [20]. Social media is also used by government bodies to provide information on where people can get help. For example, during the 2009 influenza outbreak, the Alexandria health department tweeted and texted where people could receive their H1N1 influenza vaccinations, and people flocked to the locations soon after [21]. A study from the American Red Cross shows more people are turning to social media platforms like Facebook and Twitter to learn about emergencies, get information, and check on their friends and family [22].

There is an established literature focused on studying Twitter use during disasters and specific work that explores filtering social media data during a range of natural disasters [23–25] and from different parts of the world [26]. This research includes several efforts to build pipelines that can filter through tweets and detect relevant content in real time. Studies also follow a multi-modal approach [27], using BERT for the language features and DenseNet for the image features.

Among other methodologies used, classification algorithms have been used to successfully classify tweets in 76% of the cases into low priority, such as general information about the disaster and high priority content (such as those looking for urgent [18]. Using a Markov chain, this work also estimates where the person in need of help would be situated, with an accuracy of 87% [18]. Other work uses Markov chains for user location estimation on Twitter [28]. Furthermore, communication behavior and sentiment/emotion analysis of tweets have been important to understanding the impact of natural disasters on individuals [29]. The Stanford classifier, for instance, consists of 5 categorical labels: “Very Negative”, “Negative”, “Neutral”, “Positive” and “Very Positive”. In [30]’s research, they feed collected and preprocessed tweets from three different hurricanes (Harvey, Irma, and Maria) into the Stanford classifier and it assigns one of the five categories with their confidence. Sentiment analysis can measure shifts in online users’ moods during disasters, which can then be used by authorities and triage efforts to limit damage and recover people from disasters [31].

Social media data has also been specifically analyzed during the post-disaster recovery phase of hurricanes by studying key topics that emerge in discussions between affected individuals by comparing these to discussions by those who were not affected by the disaster [32]. Twitter data has also been analyzed by studying how prevalent topics changed during the natural course of a hurricane, such as situational relief or disaster awareness [33].

### Social media integration for emergency response and disaster relief

Social media plays an important role at every stage of a disaster—from disaster preparation, coordinating responses during a disaster, and recovery in the aftermath [34]. Social media is actively used by victims during natural and other disasters [35], with some victims even using it as a coping mechanism [36]. Before their advent, other Internet-based services were used by disaster victims. For example, during the immediate aftermath of Hurricane Katrina in 2005, which primarily affected the coastal states of Texas, Louisiana, Mississippi, Alabama, and Florida, the Gulf Coast News website set up a site for people to share their hurricane experiences [37]. Pre-social media, websites, blogs, and online forums were created to help people share their experiences and ask for aid [38]; however, there was some confusion on where people should post.

During Hurricane Sandy in 2012, which affected many Atlantic-coastal states and countries, The American Red Cross utilized different social media applications (e.g. Twitter and Facebook) to carry out its mission online, which included helping the community to be more informed and prepared [39]. They did this by collating over two million online posts to review, filtering them by searching for and choosing specific keywords relevant to the Red Cross services, including “shelter” and “emotional support.” 31 volunteers responded to 2,386 of these posts, 229 posts were sent to mass care teams, and 88 posts resulted in a change in action on ground operations [39]. Because of the nature of Twitter as a traditionally public social media platform (in that, it is easy to disseminate and collect information [40]), it has grown as a platform for officials to utilize to spread awareness during disasters [41]. By utilizing content on Twitter posted during Hurricane Sandy, the research found that, while agencies utilize Twitter as a means of communication, they tend to neglect the feature that permits two-way communication, which would allow them to engage with the public more, answer questions, get further information, etc. [42]. Specifically, they found that 18% of hurricane victims without power used Twitter as their information source (as found from the study’s telephone and web-based survey results) [42]. Given the challenges of sorting and analyzing what information on Twitter is relevant and useful (an issue raised by these studies and others), developing automated emergency management tools for social media remains important.

### Advanced methodologies in disaster management using social media

The methodology proposed by [43] showcases the innovative use of social media data for tasking remote-sensing imagery during disasters. Their approach, which blends social media insights with traditional assessment techniques, highlights the importance of integrating diverse data sources for effective disaster management. This is particularly vital in large-scale natural disasters like hurricanes, where prioritizing image collection based on real-time Twitter data and integrating it with other datasets can significantly enhance situational awareness and response strategies.

A study on social media analytics in natural disaster management underscores the importance of extracting multi-dimensional data for comprehensive situational awareness. This approach aligns with the use of BERTopic for analyzing Twitter data, focusing on the spatial and temporal dynamics of hurricane-related discussions. The integration of social media data with authoritative datasets, such as census and remote-sensing data, opens new avenues for enriching analyses and enhancing the utility of social media across all phases of disaster management.

The systematic review by [44] categorizes the utility of social media in natural disaster management into six key themes: enhancing situational awareness, refining data collection methods, acting as distributed sensor systems, distinguishing between news and rumors, conducting sentiment analysis, and facilitating digital volunteerism. This comprehensive perspective on social media’s role in disaster scenarios highlights its importance in real-time communication, and information dissemination, and as a critical tool for data gathering and analysis to improve emergency response strategies.

### Utilizing Twitter for early warning and assessment

Previous work on Hurricane Sandy [45, 46] exemplify Twitter’s significant function as an early warning system. The research highlights the utility of Twitter for rapid disaster assessment and response, noting the prevalent negative sentiment during crises. This work finds that (a) combining social media data with economic losses and geo-information can provide efficient early warning and disaster assessment and (b) areas closer to the hurricane’s center or coastal regions exhibited more intense social media activity, correlating with higher damage levels, thus underlining the importance of social media in contemporary disaster management strategies [45, 46].

### Innovations in disaster assessment using social media data

By combining machine-learning techniques, specifically Latent Dirichlet Allocation (LDA), with spatiotemporal analysis, previous work assesses the impact and damage caused by natural disasters like earthquakes [47]. This method demonstrates effectiveness in identifying disaster footprints and generating damage maps in real-time, outperforming traditional models by reducing temporal lags and improving spatial and temporal resolution, thus offering a significant enhancement to current disaster management procedures.

### Supervised approaches for crisis data classification

Supervised approaches are useful, but they also are inherently limited by their reliance on supervision. Moreover, it is easy to classify something as relevant or irrelevant—a binary property. However, it becomes much more difficult as we further divide these categories. Imran et al. [48] observe that it can become increasingly more difficult as new concepts/categories outside of the umbrella emerge. Their solution required aiding supervised learning by using unsupervised algorithms to dynamically change the set of active labels as the crisis develops. Other work uses supervised machine learning methods to predict the relevance of individual tweets to hurricanes in order to 1): identify tweets that are relevant to hurricane events and 2) classify Twitter users’ evacuation behavior [49]. Their accuracy results were sufficient, with their relevance classifier achieving an F1 score of near.83.

While this helps react to shifts in categories, the actual training dataset does not grow, hence weakening the addition of new labels [48]. To resolve that problem, a new dataset would need to be created. Several tasks are involved in creating a new dataset and these also include costs of time and/or money for sourcing data, pre-processing data, defining annotation schemas, and identifying relevant words/terms (some of which are not in formal dictionaries) [50]. However, supervised learning still has tremendous potential in tweet classification, especially for sampling and filtering out tweets as part of data preprocessing [51]. However, there remains a lack of other methods of classification in this area.

### Semi-supervised approaches for crisis data classification

In addition to the challenges associated with classifying a tweet as relevant or irrelevant, veracity is another important variable. During natural disasters, it is difficult to discern veracity [52]. To combat this, stance classification is utilized. Stance classification is a machine learning model used to determine one’s attitude towards a given subject (e.g., a rumor circulating on social media). Semi-supervised approaches to determine stance classification are used due to accuracy, scalability, and speed [53, 54]. The results of utilizing semi-supervised learning models are generally as accurate, especially when datasets are further annotated. However, limitations to this methodology include the need for a set of annotated messages, which can cause difficulty in a real-time setting.

Therefore, a need exists for annotated data to achieve a semi-supervised approach. Previous work has investigated how to utilize abundant unlabeled data generated on social media by disaster eyewitnesses and affected individuals [55]. This approach shows up to 7.7% in improvement in F-1 in low-data regimes and 1.9% when using the entire training data [55].

### Unsupervised approaches for crisis data classification

The application of unsupervised approaches for crisis-related data classification has been growing as an alternative to supervised approaches which require labeled data. For example, a subset of event-relevant tweets that summarizes a real-world event such as a disaster, outbreak, riot, etc. can be discerned from Twitter in real-time using unsupervised techniques [56]. A key step of this approach is to identify similar tweets (i.e., tweets of the same type), that describe one of the highlights of the event. In a supervised setting, this would involve being able to label tweets as “landfall”, “peak”, “aftermath”, etc. This type of annotation would likely be difficult for untrained annotators as each event could have varying milestones with varying degrees of intensity. Though possible, training a model to be robust, despite heavy variation might be unrealistic.

Other work implements a Hierarchical Clustering approach and successfully utilizes Named Entity Recognition (NER) and a Parts of Speech Tagger (POS) for event extraction and sub-event identification from Twitter feeds [57]. Event identification and discerning relations is important for information retrieval, which in turn, can be used in disaster management to better plan response operations for future events [57]. POS is a tool in unsupervised machine learning and natural language processing that attempts to understand the grammatical structure of a sentence and to disambiguate words that have multiple meanings by assigning them a part of speech, such as a verb, noun, subject, object, etc. NER, a tool used in unsupervised machine learning and natural language processing, extracts and categorizes information from text. For example, NER is used to identify colors, people, seasons, occasions, dates, etc.

One popular method utilizing unsupervised learning is the Latent Dirichlet Allocation (LDA) technique, which in disaster-related communication on Twitter, can identify latent categories of tweets such as affected individuals and disrupted services [58]. LDA is a topic modeling technique that will be explained more in the next section, along with some of its applications in other research.

### Topic modeling

Topic modeling is a subset of text analysis methods. The approach seeks to categorize words into particular themes by clubbing them together based on statistical probabilities or algorithms. In many topic modeling approaches, clustering algorithms assign content (e.g., words, phrases, articles, or posts) to a particular cluster. Topic models are mixture models, which means that every item will be given a probability of belonging to a particular theme, or topic. Sophisticated iterative Bayesian techniques are usually used to determine this probability.

As a solution, some have leveraged LDA, an unsupervised clustering algorithm [59], to topically cluster key points of a crisis event [56]. There are many topic modeling methodologies in the area of natural language processing. LDA is one of the first such topic models. It is an unsupervised learning-based approach. LDA views documents as a bag-of-words; therefore, it does not consider order. For each document *d*, we assume that there are *k* different topics. These *k* topics are distributed as an *α* distribution across the document. *α* is the first hyperparameter of an LDA model, which controls the expected number of topics in a document. The second hyperparameter *β* is the distribution of words per topic in the document. Finally, we have our previously defined *k* number of topics as the third hyperparameter. Significant human labor hours are saved in developing unsupervised algorithms, as mentioned previously. Additionally, unsupervised methods, because of their iterative behavior, are better for applications best computed in real-time, (e.g., disaster management).

When clustering Twitter data, similar tweets are brought together while dissimilar tweets are pushed apart. Such methods allow for the identification of subgroups without prior information about these subgroups. However, unlike supervised learning, such methods tend to soft-cluster tweets and a single tweet could be assigned to multiple topics. There are some challenges of using LDA to discern meaningful topics when LDA is applied to a noisy data set [60]. However, most news articles and Twitter posts will contain more information than just the key events (e.g., historical information, commentary, sponsorships, etc.). This can exacerbate overlap between topics. As with supervised learning, pre-processing data is critical. Substantial work exists which has used LDA to perform event detection amongst tweets [48, 50]. Previous work has experimented with three clustering algorithms: LDA, Nonnegative Matrix Factorization (NMF), and k-means [61]. Out of the three options, LDA had the highest accuracy in clustering together signal tweets out of a highly unbalanced dataset containing signal and noise tweets with noise tweets as the majority [61].

While these approaches focus on a single crisis event, LDA can also be used to compare clusters of signal and noise across multiple events and timelines. In previous work, LDA was used to study differences between multiple hurricane events, but only resulted in limited evidence of differentiability as measured by Hellinger’s Distance [13]. However, with cleaner data, it might be possible to achieve more conclusive results. In addition to comparing across the entirety of multiple datasets, previous work has been done in comparing subsections of a dataset across time. Zhou et al. [62] define a system that can summarize the timeline of high-impact events in real time on Twitter. They propose that this system could be applied as a “replacement of a manually generated timeline and provides early alarms for disaster surveillance” [62]. A similar technique could be applied to breaking down the chronological stages of a hurricane and detecting and responding to users in need before they reach emergency conditions. However, there is a scarcity of work that leverages topic modeling to compare different stages across multiple hurricanes to identify quantifiable differences.

Previous work has established the utility of studying natural disaster-related tweets using topic modeling (often by discerning which topics were expressed by particular groups of people such as victims, disaster relief workers, government agencies, etc.) [47]. However, much of this work has generally been built around older iterations of LDA. Though newer topic modeling approaches with much larger embeddings, such as BERTopic [63], have been extensively used in the COVID-19 pandemic, there is a dearth of applying such methods to natural disasters. In the context of COVID-19, BERTopic was successfully used to identify topic clusters of racist tweets towards Asian people [64] and to identify six thematic emotional areas (e.g., fear) experienced by people during the pandemic [65]. In the context of vaccine hesitancy, this topic modeling approach was successfully used to find political biases within pro- and anti-vaccine communities [66] and was deployed to identify themes in QAnon conspiracy-related content [67].

### Overview of previous work

Previous work undertaken in this area is summarized in S1 Table. Because significant differences in terms of methods, data, strengths, and findings exist in the literature, S1 Table provides an accessible method to comprehend the variations among different works in the literature.

## Methods

### Data collection and processing

S1 Fig depicts the overview of our unsupervised approach pipeline. As the hurricanes we study span seven years, we utilize diverse data sets, which were collected using heterogeneous methods (See Table 1). We collected data from the Twitter API in different ways because at the time of Hurricane Sandy, we deployed specific keywords to search Twitter, and by the time of Hurricane Harvey, we had a proactive Twitter data collector already in operation. We utilized a third-party tool, because we did not have access to our proactive Twitter Spritzer data collector in 2018. The data collected were for U.S. hurricane events and included Hurricane Sandy (Sandy), Hurricane Harvey (Harvey), Hurricane Florence (Florence), and Hurricane Michael (Michael). These terms are words associated with each hurricane that refer to the most common, non-article-related words found in tweets from each of the hurricanes. We identified these terms through topic clustering with the LDA model and varying the size of the “num_topics” parameter to see which would produce the best set of topics per dataset.

**Table 1:** Summary of datasets used; duplicate tweets removed.

Hurricane	Year	Corpus Size	Mode of Collection
Harvey	2017	102,904	Twitter Spritzer API
Sandy	2012	85,322	Twitter REST API
Florence	2018	862,595	Twitter REST API (Netlytic)
Michael	2018	696,224	Twitter REST API (Netlytic)

To identify the terms most associated with each hurricane, we applied Latent Dirichlet Allocation (LDA) for topic clustering. During this process, we varied the “num_topics” parameter, testing values ranging from 5 to 50 in increments of 5, to find the most suitable number of topics for each dataset. We evaluated the quality of the topics using coherence scores, a metric that assesses the interpretability and relevance of the topics generated. After experimentation, we determined that the optimal number of topics varied by dataset: Sandy produced the best results with 20 topics, Harvey with 25, Florence with 15, and Michael with 10. These values provided the most distinct and meaningful sets of topics for each hurricane, ensuring that the terms identified were representative of the key themes within each dataset.

Data for Sandy was collected in real-time via calls to the Twitter REST API using PHP scripts. For Florence and Michael, data was collected via Netlytic [68], a third-party software tool that made calls to the Twitter REST API in 15-minute intervals using various manually-selected hashtags (e.g., #Florence) and keywords (e.g., “storm”). Lastly, data for Hurricane Harvey was collected using a Python-based script that was collecting data from Twitter’s ‘Spritzer’ STREAM API, which collects 1% of all tweets regardless of keyword, hashtag, etc. Hurricane-related search terms were applied post-facto to extract relevant data for Harvey. Each of these methods of data collection resulted in accumulating a significant number of non-relevant tweets alongside tweets relevant to the hurricane, which is consistent with established hashtag and keyword data collection methods [24, 62, 69].

### Pre-processing

We preprocessed the data by first removing links from the tweets since they are not relevant to topic modeling. We removed retweets as well as duplicated punctuation and numbers that occur in tweet text. Words with a length of less than 2 or greater than 11, as well as emojis, were also removed. This range was chosen based on some common social media grammar rules. For example, on Twitter, some users will not use spaces in between each word, making these posts unusable for our research. We also ensured that there were no retweets or quote tweets included in the collection. Lastly, we removed stop words using the stop words list from NLTK.

Lemmatization and tokenization were not performed. This choice was made to maintain the original structure of the tweets, which allowed the topic modeling to capture more nuanced patterns in the data. By not applying lemmatization, we retained the morphological forms of words, which could provide additional context in the clustering process. We utilized stop words from the NLTK library to remove common, non-informative words from the dataset. While the standard NLTK stop word list was sufficient for general cleaning, we recognize that in specific contexts, additional custom stop words might be necessary. For example, terms like ‘China,’ ‘Hong,’ and ‘Kong,’ which appear under the ‘Select Words Grouped by BERTopic’ categories in Table 3, were not removed, as they are not included in the NLTK stop word list. These terms were retained because they hold significant relevance in the geographic and thematic analysis within the topic modeling process.

### Temporal frequency analysis

We produced timeseries plots for Hurricanes Harvey, Sandy, Florence, and Michael. This was done by creating custom scripts, which first cleaned the data sets for each hurricane and then filtered, grouped, and counted the data. This was done to determine the corresponding number of tweets for each phase of each Hurricane (i.e., Warning, Recovery, and Response). To render the timeseries plots, we utilized ggplot2 [70], an open-source visualization library for R Studio, to represent the tweet frequency per phase of each hurricane.

We examined the social dynamics surrounding four major hurricanes: Harvey, Sandy, Florence, and Michael. To understand public discourse during these events, we created custom scripts to clean and prepare our data. We filtered, grouped, and analyzed each tweet to capture the public’s response. Our goal was to trace the public conversations from the initial warnings, recovery efforts, and community resilience. By documenting Twitter activity throughout each hurricane’s lifecycle, we identified shifts in sentiment and engagement during different phases. To present our findings, we used visualization libraries to create detailed time series plots. These plots represent tweets that depicted the evolving narratives around each hurricane. Our data-driven approach was used to highlight societal responses and emerging patterns during these natural disasters.

### BERTopic

For this study, we evaluated several topic modeling approaches discussed previously. We ultimately selected BERTopic for its extensibility and its successful use in previous work [71]. The authors of the Bidirectional Encoder Representations (BERT) [72] introduced the transformer-based architecture in language models, resulting in better results for downstream NLP applications. A transformer is an attention model that contains two parts: an encoder and a decoder [73]. The encoder reads the input text and the decoder predicts the text. To generate language models, BERT only requires the encoder. The encoder reads the entire sequence of text at a time, not in a particular left-to-right or right-to-left direction, which gives BERT its bi-directionality. This is advantageous because the model can take in the context based on the surrounding words.

BERTopic specifically leverages this characteristic of the BERT language model. It uses the term frequency-inverse document frequency (TF-IDF) to translate input documents into clusters. Embeddings are reduced using UMAP in the BERTopic Python library. This is a general-purpose manifold learning and dimensionality reduction algorithm. When BERT embeddings are used, the dimensionality of the vectors is very high, which results in a lot of compute resources being used. There are several benefits of dimensionality reduction, such as: improving data quality, simplifying the process of clarification, improving efficiency, removing noisy or redundant data, and more [74]. Our work provides evidence for the success of topic models such as BERTopic as a data source to identify significant differences between signal and noise for hurricane events. Hierarchical Density-Based Spatial Clustering of Applications with Noise (HDBSCAN) [75], a hierarchical clustering algorithm, is then used because noise is then modeled directly as outliers and prevents unrelated documents from being assigned to any cluster. This approach has been used to better topic representations [63].

We imported datasets related to hurricanes Harvey, Sandy, Michael, and Florence, and combined them into a single DataFrame. Then text data from these hurricanes was extracted into a list. Our environment was Conda (rapids-22.04) and we installed necessary libraries, including BERTopic, gensim, and kaleido. To undertake topic modeling with BERTopic, we created an instance of the UMAP model with parameters “n_components” = 5, “n_neighbors” = 15, and “min_dist” = 0.0. Then we created an instance of the HDBSCAN model with parameters “min_samples” = 10 and “gen_min_span_tree” = True. These models were passed to BERTopic for topic modeling. In terms of tuning the HDBSCAN parameters, the “min_samples” parameter in HDBSCAN (which determines the minimum numbers of points required to form a dense region) was set to 10, further improving the robustness of the clusters. The “gen_min_span_tree” = True parameter was used to generate a minimum spanning tree for visualization of the hierarchy of clusters. We fit the BERTopic model on the combined hurricane text data “english_text” and extracted the topics and their probabilities. We saved the resulting topic information to a CSV file for analysis.

### GPU acceleration

The previously mentioned UMAP and HDBSCAN algorithms are extremely compute-heavy due to the complex calculations carried out on our large corpus of over 1.7 million tweets. Hence, we use a GPU Accelerated version of these algorithms and then use the outputs in BERTopic. This speeds up computing by more than 20x, reducing the training time from over 40 hours to approximately two hours. We use the cuML suite of python libraries [76]. We parallelized these UMAP and HDBSCAN functionalities using CUDA programming on GPUs to maximize computing efficiency.

To efficiently process the large-scale textual data and speed up the topic modeling task, we leveraged GPU acceleration in our BERTopic implementation. Specifically, we utilized the RAPIDS suite of libraries [77], which includes GPU-accelerated versions of UMAP and HDBSCAN, crucial components in our topic modeling pipeline. Our experiments were conducted on a machine equipped with an NVIDIA Tesla V100 GPU with 32GB VRAM and an Intel Xeon CPU with 128GB of RAM, running Ubuntu 20.04. The software environment was configured with Python 3.9 and included the following key libraries: ‘cuml 22.04’ for GPU-accelerated UMAP and HDBSCAN, ‘bertopic 0.9.4’ for topic modeling, and the ‘gensim’ and ‘kaleido’ libraries for text processing and visualization. Though previous work reports up to 20x improvements with GPU acceleration [78], we quantified the performance gains achieved through GPU acceleration via a control experiment comparing the training time of the BERTopic model on both GPU and CPU. The same model configuration and dataset were used in both scenarios, with the only difference being the underlying hardware. In the GPU-accelerated environment, the time required to train the BERTopic model was significantly reduced. For comparison, we also ran the same BERTopic model using CPU-only versions of UMAP and HDBSCAN to measure the difference in performance. We found that the GPU-accelerated BERTopic model completed the training in approximately two hours, compared to over 40 hours required by the CPU-only implementation. This represents an acceleration of more than 20x, highlighting the effectiveness of GPU acceleration in handling large-scale topic modeling tasks and is in line with previous work. The significant reduction in processing time not only makes large-scale topic modeling feasible, but also enables iterative analysis, which ultimately contributes to more refined and robust results.

## Results

### Topic coherence and correlation

There are several quantitative ways to evaluate topic models. Coherence Tests are used to evaluate topic models, and to find the optimal number of topics. We use the coherence measure from Roder et al [79], which is the product of a four-stage process. First, the documents are segmented. We found the highest coherence value = 0.67 to be present for 20 topics. Therefore, we conclude that this is the optimal number of topics that our model should cluster our corpus into. There are other established topic correlation models. One such application for topic correlation in multimedia information retrieval includes using a bag-of-features model for images and a topic distribution for the text component [80]. Another application of topic correlation is for ranking topics based on informativeness in the clinical field [81].

The correlation values between the topics are presented as a correlation matrix in S2 Fig. This figure shows that the topics are minimally correlated with one another and are separated as they belong to distinct phases. As S2 Fig illustrates, most topics have unique content and little to no overlap amongst the different topics.

### Temporal frequency analysis

Table 2 provides a summary of our temporal frequency analysis. Each hurricane is represented by a row in the table and the values in the three columns (i.e., Warning, Recovery, and Response) each contain the frequency of tweets, and the percentage of tweets the frequency represents are included in parentheses. To conserve space in the main body of the manuscript, we provide the time series plots for each hurricane in the S3–S6 Figs. That being said, Table 2 succinctly compares the frequency of tweets by phase per hurricane.

**Table 2:** Tweet frequency by phase (percent by phase in parentheses).

Hurricane	Warning	Recovery	Response	Total
Harvey	7,797 (7.58%)	42,925 (41.71%)	52,182 (50.70%)	102,904
Sandy	296 (0.35%)	17,629 (20.66%)	67,397 (78.99%)	85,322
Florence	106,152 (12.31%)	284,957 (33.03%)	471,485 (54.66%)	862,595
Michael	148,489 (21.33%)	289,800 (41.62%)	257,935 (37.05%)	696,224

Hurricane Harvey saw the most tweets derived from the response phase and frequency peaked during the recovery phase (see S6 Fig and Table 2), which saw its peak at just under 8,000 tweets. Following the response phase for tweet frequency is the recovery phase, and finally, the warning phase (see Table 2). Hurricane Sandy saw the most tweets derived from the response phase (see Table 2) and frequency peaked during the recovery phase (see S5 Fig), which saw its peak at over 30,000 tweets (see S5 Fig). Following the response phase for tweet frequency is the recovery phase, and finally, the warning phase, which had only a handful of tweets (see Table 2).

For Hurricane Florence, the response phase produced the most tweets. However, the warning phase peaked at over 85,000 tweets (see S4 Fig). Following the response phase for tweet frequency was the recovery phase and, lastly, the warning phase (see Table 2). For Hurricane Michael (see S3 Fig), the recovery phase produced the most tweets, though the warning phase peaked at over 90,000 tweets (see S3 Fig). We found that following the recovery phase for tweet frequency was the response phase and, lastly, the warning phase (see Table 2).

As Table 2 and S3–S6 Figs illustrate, the frequency of tweets by most frequent phase did vary in the case of these four hurricanes. However, in our temporal frequency analyses, we found that for Hurricanes Florence and Michael, the warning phase represents the peak number of tweets, while for Hurricanes Sandy and Harvey, this is represented in the recovery phase. The response phase had the highest frequency for any phase for Hurricanes Harvey, Sandy, and Florence. Hurricane Michael was the exception as its recovery phase produced the most tweets.

### Thematic analysis

Once topics are generated by BERTopic, human interpretation of them is generally needed. This section details the results we derived from the data sets we studied as well as observing some of the similarities and differences we deciphered between hurricane events. Following the suggestion of Kar and Dwivedi [82] that “topic models derived after text summarization can be mapped to constructs or themes” to assist with theory building, we attempt to infer important themes from our topic modeling results to better contribute to the literature. We first explain our results derived from topic modeling and then discuss our results from the modeling and coherence tests.

Communication patterns have been analyzed thoroughly in social media [83] based on topics obtained by clustering algorithms. Understanding these communication patterns has the potential to help authorities use this information to send targeted alerts or otherwise make crisis communication more effective in other disasters. Based on the most common words clustered as topics by BERTopic, we can distinctly classify thematic constructs by three major hurricane phases—warning, recovery, and response [83]. Table 3 illustrates the inferred topic results that we developed from aggregated datasets. We analyze tweets from four combined hurricane-related datasets: Harvey, Sandy, Michael, and Florence. We ignore all outliers produced by the model. Our discussion seeks to unpack the significance of the inferred topics we derived. Given that BERTopic represents an unsupervised method, it is important to confirm the human interpretability of these results about areas of thematic importance to relief organizations, first responders, and other stakeholders.

**Table 3:** Temporal phase and thematic results by topic and percentage of tweets.

Phase	Theme	Select Words Grouped by BERTopic	Percent	Tweet Example
**Warning**	User Concern—Religious	temple, faith, lord, visits, church, meals, god	8.43	“Pets need love too! Here’s how to prepare and protect your furry friends ahead of #Harvey”
	Weather Conditions	winds, storm, turn, plane, strong, land, batter	7.67	
	Locations	china, hong, kong, luzon, worlds, hill, signal	5.82	
	Preparedness	tips, life, lives, drive, wind, inches	4.72	
	User Concern—Informational	info, views, news, call, ncsc, nasa	4.47	
	Storm Watch	miles, mph, nhc, winds, coast, max, outer, space, lens	3.06	
**Recovery**	Relief	giving, cash, time, women, amplify, join, border, ngos, source	10.25	“ALERT! #HoustonFLOODS MOM W/ 2 KIDS IN HIGH WATERS! Here’s address PLZ RETWEET FOR HELP! #Harvey #HELP #NOW”
	Aftermath	drone, beach, deadly, invoke, turned, shows	5.17	
	Damage	puerto, rico, die, island, ricans, hit, died, people, left	5.06	
	Disaster Effects	city, tore, relief, yet, amp, amplify, since, seen, water, morn	3.59	
	Recovery	done, battle, means, stamp, stay, still, knew	3.53	
	Emotional Toll	cries, tears, dies, enter, tilt	3.26	
	First-hand Accounts	wish, look, past, flying, scene, plea, scene, headed	3.04	
**Response**	Officials	state, gov, term, change, banned, government, hit	6.42	“Harvey recovery continues as President Trump heads down to visit battered #Texas and Louisiana”
	Power outage	lose, power, stage, awful, worry, snow, winter, storm	5.73	
	Entertainment	bts, btstwt, kpop, event, cinema, film, world, gma	5.06	
	Death Tolls	killed, people, year, case, died, storm, rather	4.66	
	Pets/ Animals	hurricane, category, climate, victims, animals	3.64	
	Questions/ Concerns	asked, course, mostly, lake, nobody, first, second, fourth	3.19	
	Retail	ads, began, says, bought, worth, since, turns	3.18	

#### Patterns of warning phase

Our thematic results by temporal phase (see Table 3) were clustered by our BERTopic model and have a distinct vocabulary with the warning phase, which we were able to successfully discern into six different themes. During the onset of a hurricane, people are, unsurprisingly, concerned about weather conditions and tracking the storm. We find that words like ‘winds’, ‘mph’, ‘miles’, and ‘strong’ belong to these themes and in turn, the documents belonging to these distinct topics are within the warning phase. We also see topics pointing to two types of user concern—one religious and one informational. The religious user concerns theme contains words about ‘temple’, ‘church’, ‘faith’, and ‘god’. A different cluster also contains documents with the theme of informational user concerns containing words like ‘info’, ‘news’, and ‘views’. Another important cluster that we found relates to the preparedness for the onset of a potential disaster. Words such as ‘tips’, and ‘drive’ indicate that the documents represent preparation or advice as a part of the warning. Interestingly, the most prevalent theme was religious concerns, while the least prevalent theme was storm-watch tweets. Other themes in the middle mainly focused on preparing other people for the storm, which included sharing locations, conditions, and other information.

#### Patterns of recovery phase

Social media content during landfall represents a diverse set of content. Specifically, individuals are looking for help, posting about damage, and actively seeking to recover from the storm. Our BERTopic model successfully sorted topics into these distinct themes—disaster relief (highest prevalence), damage/aftermath, and recovery, themes extended from previous work by others [83]. Within disaster relief, people are searching for help as well as people offering to help. Although we do not see a very strong distinction, there is a discernible topical difference between the two. If examined together, this allows platforms to connect those looking for help with those offering help, particularly in cases where officials are not able to respond immediately.

#### Patterns of response phase

The aftermath (response) phase is composed of post-landfall themes and particularly relates to how individuals are responding to hurricane events. This could be particularly useful to disaster management organizations in their decision-making of where to deploy resources. Our BERTopic model identified 7 topics out of 20 as being a part of the response phase. Power outage is often a common outcome of natural disasters and we see a cluster of words like ‘lose’, ‘power’, and ‘awful’ within this topic. People often tweet about officials and look for information from them. We see clusters about officials and questions respectively. Death tolls are another important cluster that our model can derive, consisting of words like ‘killed’, ‘died’, ‘case’, and ‘people’. The most prevalent topic was about officials, while the least prevalent topic was retail. There were also tweets about entertainment, pets and animals, and other themes.

## Discussion

To understand disaster communication patterns, our study discerns tweet distributions over time and by theme during different hurricane phases—warning, response, and recovery. Though previous work (e.g. Belcastro, et al. [84] focuses on detecting sub-events as secondary disaster effects by utilizing geotagged social media posts to identify localized incidents, we uniquely employ BERTopic to categorize themes without incorporating spatial data. Our analysis is also broad, covering over 1.7 million tweets from hurricanes Harvey, Sandy, Florence, and Michael. Previous frameworks such as SEDOM-DD [84] enriched non-geotagged posts for precise sub-event localization and the identification of specific incidents. We extend and develop from such approaches by opting for a focus on temporal and thematic constructs rather than geographical emphasis. Our study therefore uniquely contributes to the literature by highlighting thematic variations across hurricane phases, which we hope will aid disaster management organizations in understanding public communication patterns.

## Future work and limitations

We perform coherence tests to identify the optimal number of topics that BERTopic should cluster our corpus into. We found that identified topics corresponded to three broad hurricane phases—warning, response, and recovery. However, future work could employ other categorization methods for topic optimization. We contribute a GPU-trained topic model on hurricane data which can be used by others for classification of data. Future work could also use our parallelized approach with data from more hurricanes to see if the thematic areas we discerned remain consistent.

One of the limitations of our approach is that determining themes from hurricane-related text data contains ambiguity and uncertainties. Moreover, even if a human annotator determines that a tweet is relevant, the ground truth may not be discovered until a rescuer or other stakeholder is literally on site. Furthermore, given the incidence of bots, misinformation, and disinformation on Twitter [85], ascertaining the veracity of a tweet continues to pose significant challenges for crisis informatics work that uses social media data. Any application of our approach, therefore, would need to be tested by stakeholders on the ground to determine if tweet content marked as urgent or those seeking assistance is reflective of a real situation on the ground. Also, the methods by which we collected tweets for each hurricane varied. As a result, this has a potential impact on our findings and the type of tweets collected. This could also impact the way different phases of the hurricanes were perceived by victims and responders.

Another limitation to highlight is that we identified tweets regardless of location. Future work would benefit from spatial analysis (e.g., via geolocation), which could provide extremely useful information to first responders, disaster management organizations, and other stakeholders. Data collection of tweets in future work would benefit from specific optimization for inferring location (as we focused purely on textual content and, of course, not all of our collected tweets were posted by disaster victims). A further limitation is that searching for tweets based on names of hurricanes (IE: “Sandy”, “Katrina”, etc.) can be an insufficient means of data collection [86]. Though we did use diverse keywords besides hurricane names, future work could involve even more keywords (e.g., more location-based keywords to further reduce irrelevant data).

An important conclusion of our analysis is that a large set of tweet data from very different hurricanes does have similarities in terms of tweet-derived themes. However, a limitation of our approach is that it does not take into account differences in demographics such as age, race/ethnicity, and the education level of those tweeting. Rather, we are aggregating all tweets together by all users. The preparedness of local populations is a variable we also do not consider. Future work could use samples of tweets stratified by these variables to conduct comparative topic model-based analysis.

## Conclusion

Given that social media platforms have emerged as alternate venues for people to request help in the US during natural disasters, our study sought to evaluate methods for identifying relevant content from these platforms. A limitation of supervised approaches is their dependency on human annotators that train the model regarding which posts are relevant and which are not. We developed a successful model without human annotation, which can automatically cluster hurricane-related tweets thematically. This pre-trained model can be used to predict various hurricane-related document classifications. We use Twitter data from four US hurricane events over seven years to explore whether: (1) there is a quantifiable difference between tweets relevant to hurricane-related disaster relief and all other tweets, and (2) whether it is possible to consistently differentiate between tweets from different stages of hurricanes. We employ BERTopic, a transformer-based topic model, to answer these questions and identify topics across multiple hurricanes.

We find that during all stages of hurricanes, themes can be successfully discerned using our approach. Another contribution of our study is an evaluation of how tweet content varies significantly between different stages of hurricanes. The success of our BERTopic model also indicates that high performance with large, high-quality datasets (in our case, over 1.7 million unique documents) is possible with a GPU-based pipeline. Ultimately, our study establishes an evidence base for the utility of topic models to detect consistent textual trends in hurricane-related social media data. Moreover, we demonstrate both the success and utility of clustering tweets using a transformer-based approach. Disaster management organizations can use our approach as well as data to gain a greater understanding of the types of themes social media users are likely to be producing and consuming during hurricanes and what phases of a hurricane these correspond to. By further understanding these themes, disaster management organizations may be better equipped to deal with the specific, unique physical and emotional needs of disaster victims. Social media, specifically Twitter, has also been utilized as a means of increasing confidence in emergency management institutions [87]. From a practical perspective of transitioning our findings and approach for the benefit and use of those on the ground managing a natural disaster, our framework could be specifically extended and developed by others into an interactive dashboard or other tool customized to stakeholder needs. For example, a BERTopic-based model drawing data from hurricanes over time could be integrated into existing natural disaster dashboard systems [88, 89].

## Supporting information

S1 Table Comparative table of manuscripts mentioned and our framework (Last row). (TIF)

S1 Fig Pipeline of our unsupervised learning approach. (TIF)

S2 Fig Matrix displaying the similarity of themes within our analyzed tweets. (TIF)

S3 Fig Time series plot showing the frequency of tweets for Hurricane Michael per phase of the hurricane. (TIF)

S4 Fig Time series plot showing the frequency of tweets for Hurricane Florence per phase of the hurricane. (TIF)

S5 Fig Time series plot showing the frequency of tweets for Hurricane Sandy per phase of the hurricane. (TIF)

S6 Fig Time series plot showing the frequency of tweets for Hurricane Harvey per phase of the hurricane. (TIF)
