# Supplementary figures and images for: Examining hurricane–related social media topics longitudinally and at scale: A transformer-based approach
Source: PLoS One. 2025 Jan 24;20(1):e0316852. doi: 10.1371/journal.pone.0316852 (PMC11760010; doi:10.1371/journal.pone.0316852)

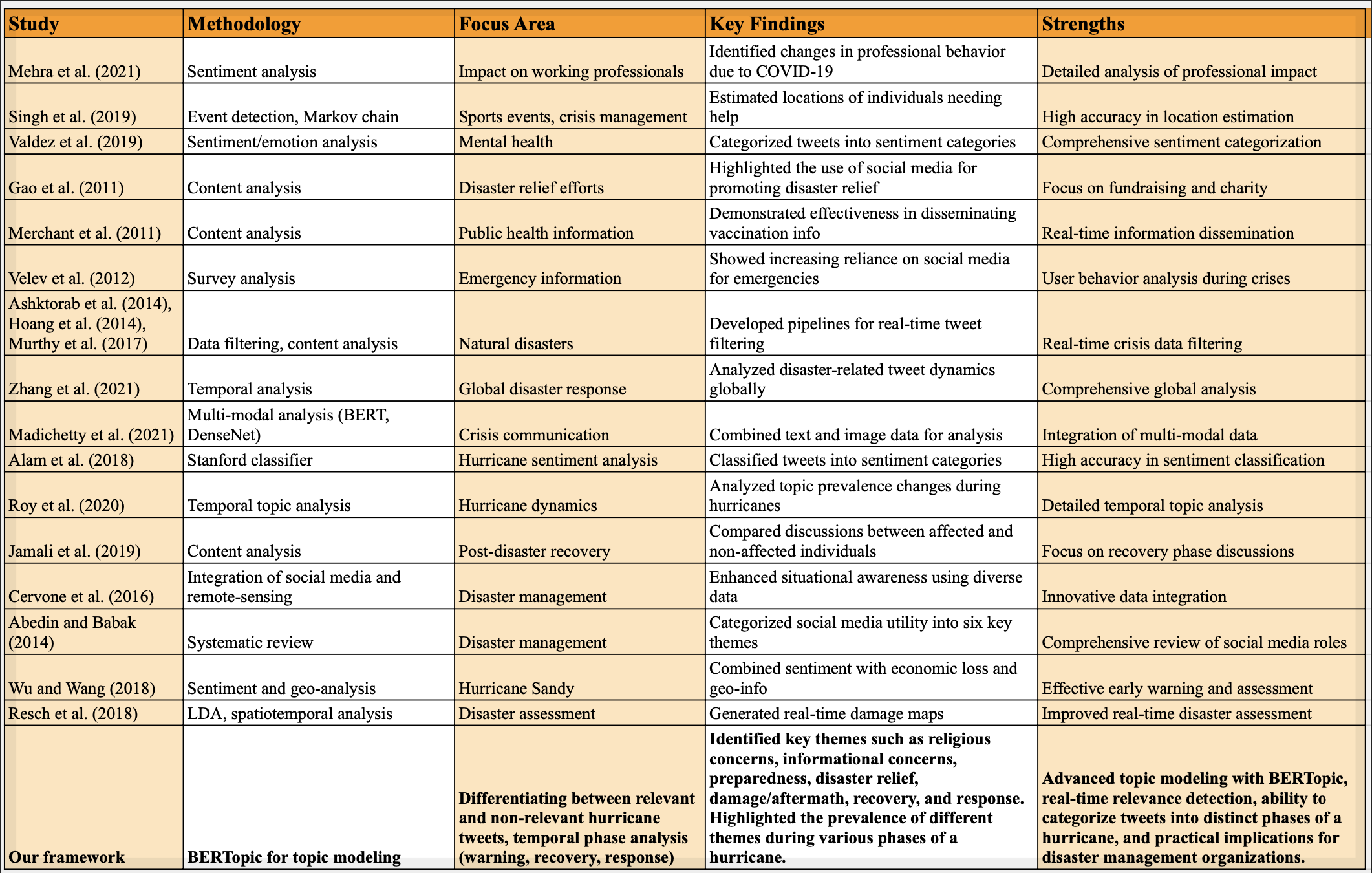

Supplement: S1 Table — (TIF) [file pone.0316852.s001.tif]

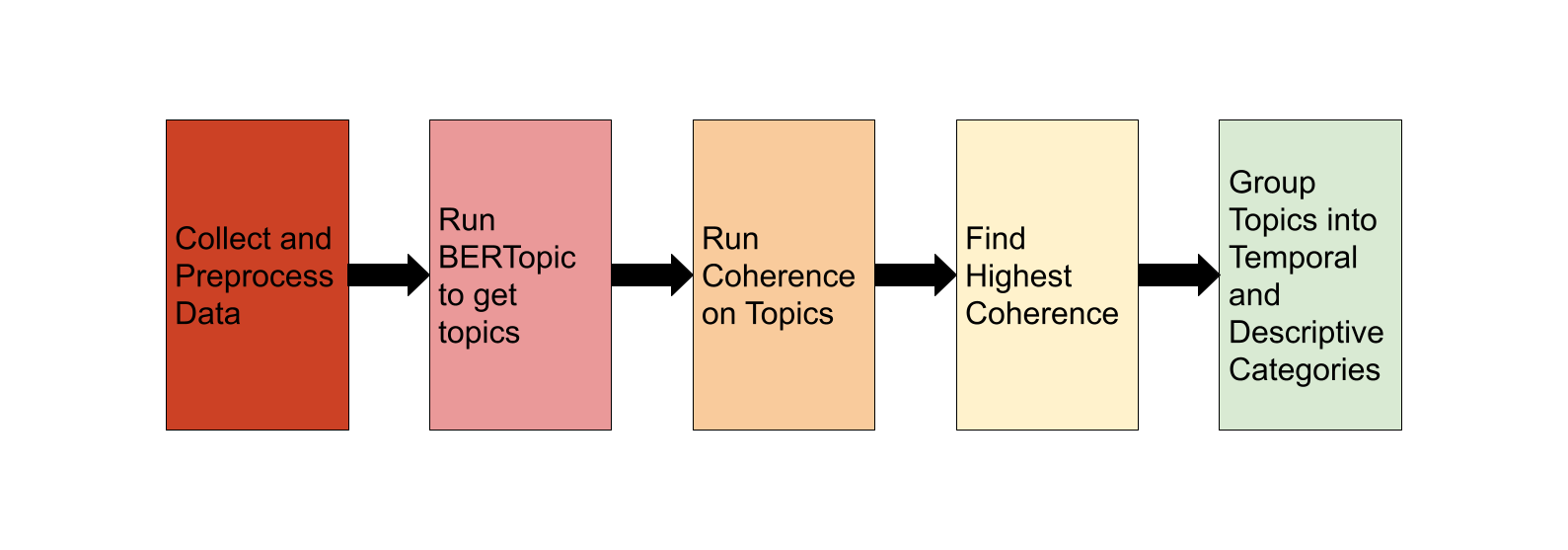

Supplement: S1 Fig — (TIF) [file pone.0316852.s002.tif]

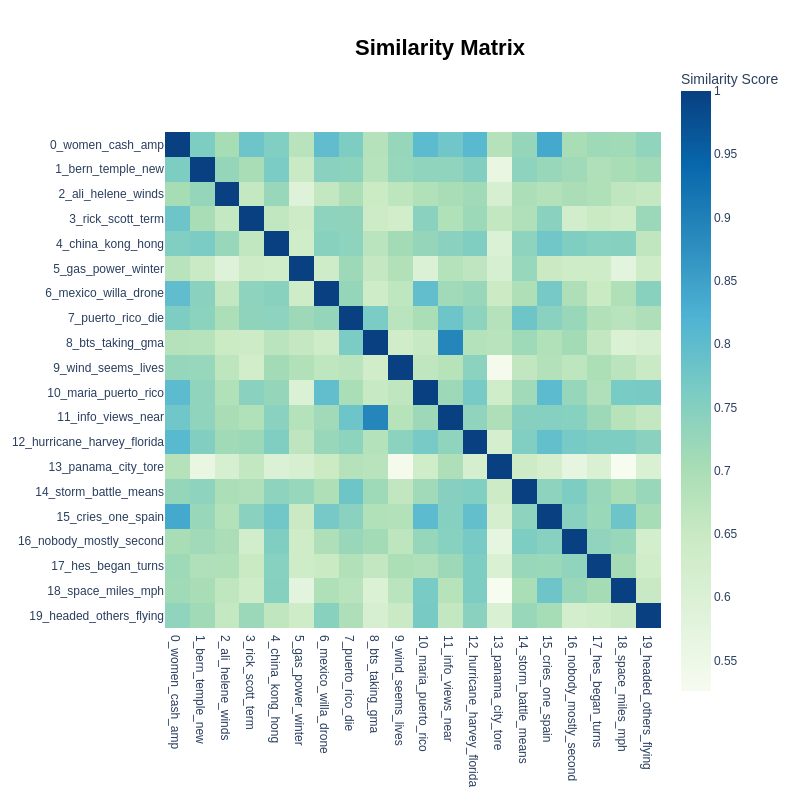

Supplement: S2 Fig — (TIF) [file pone.0316852.s003.tif]

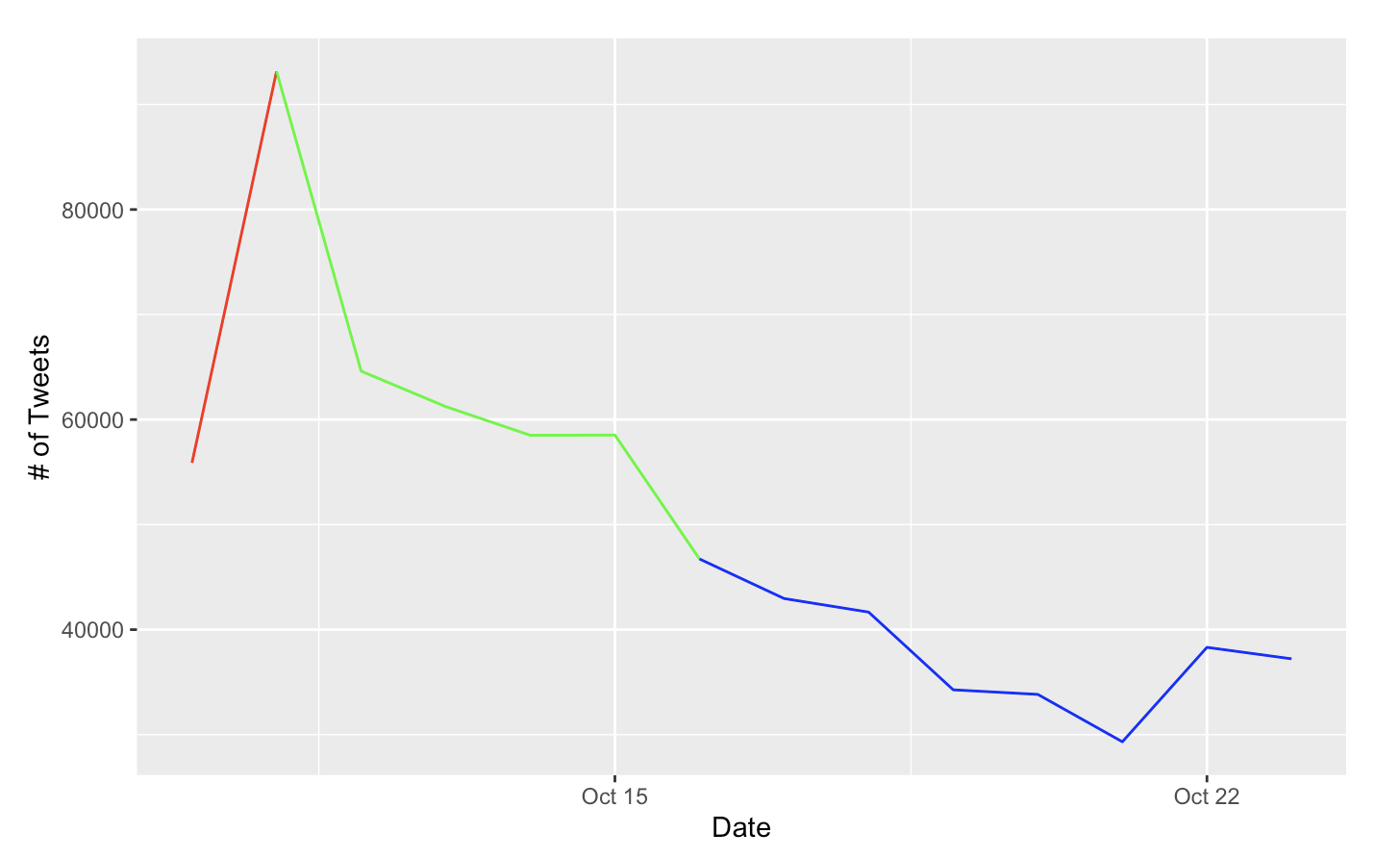

Supplement: S3 Fig — (TIF) [file pone.0316852.s004.tif]

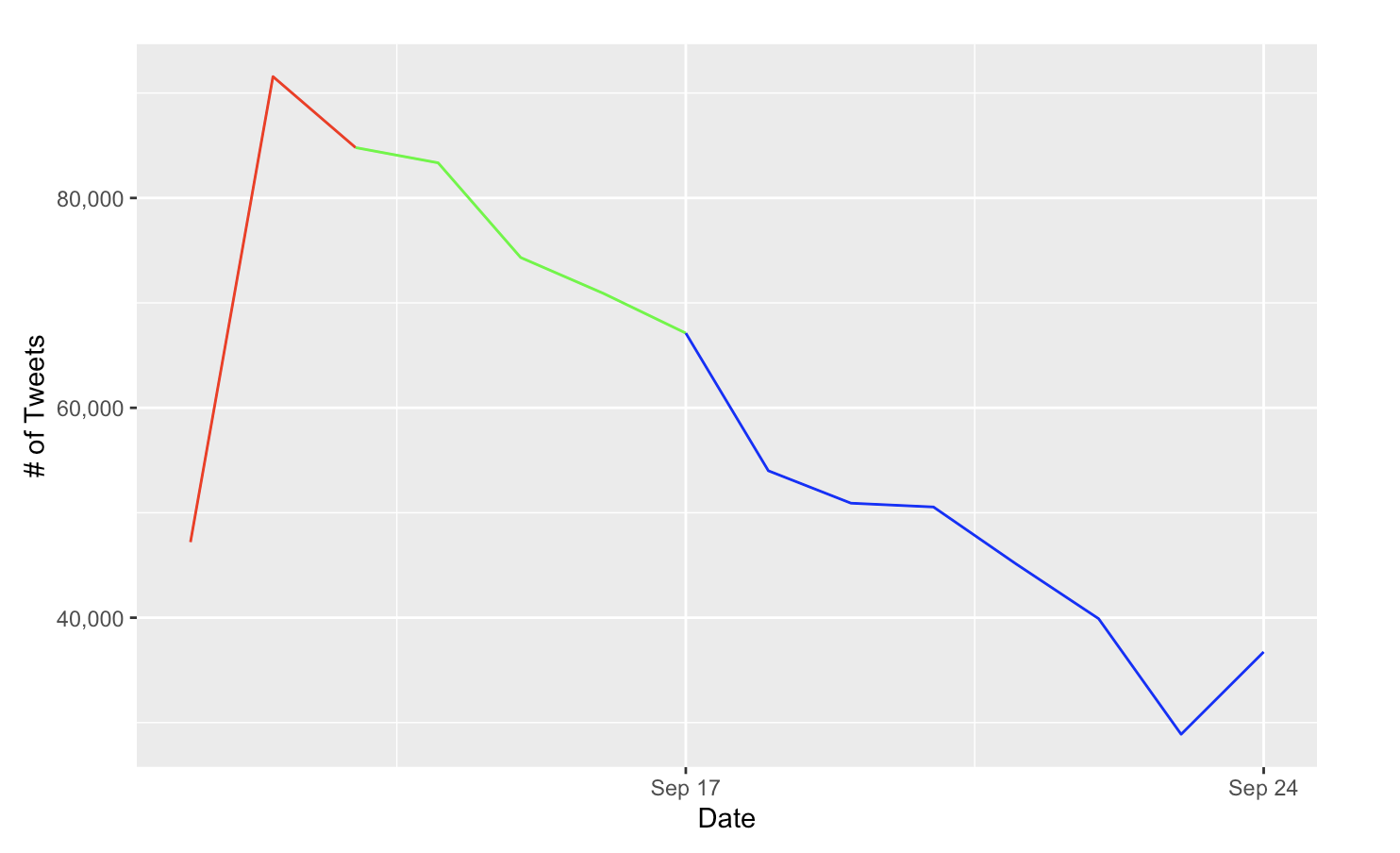

Supplement: S4 Fig — (TIF) [file pone.0316852.s005.tif]

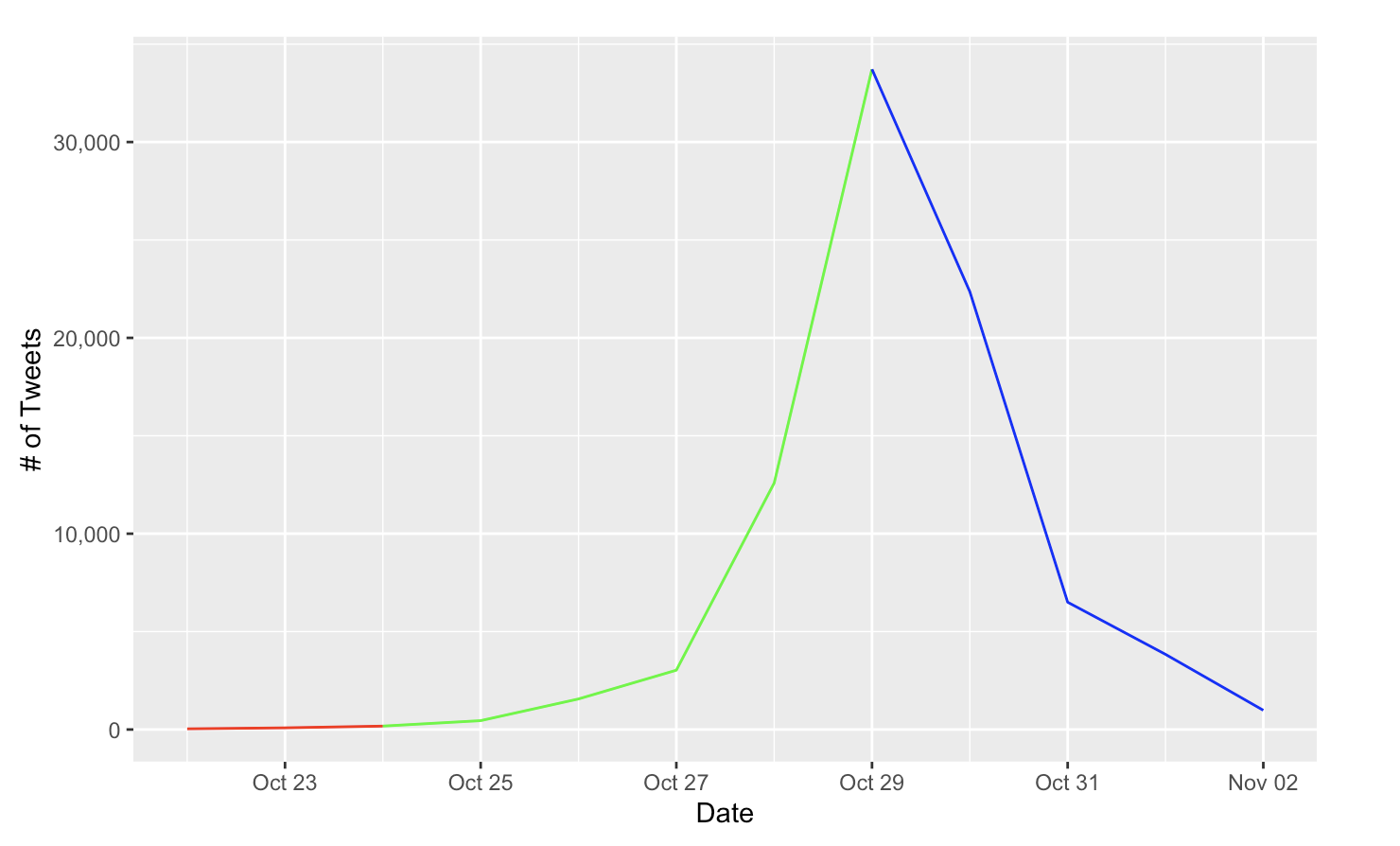

Supplement: S5 Fig — (TIF) [file pone.0316852.s006.tif]

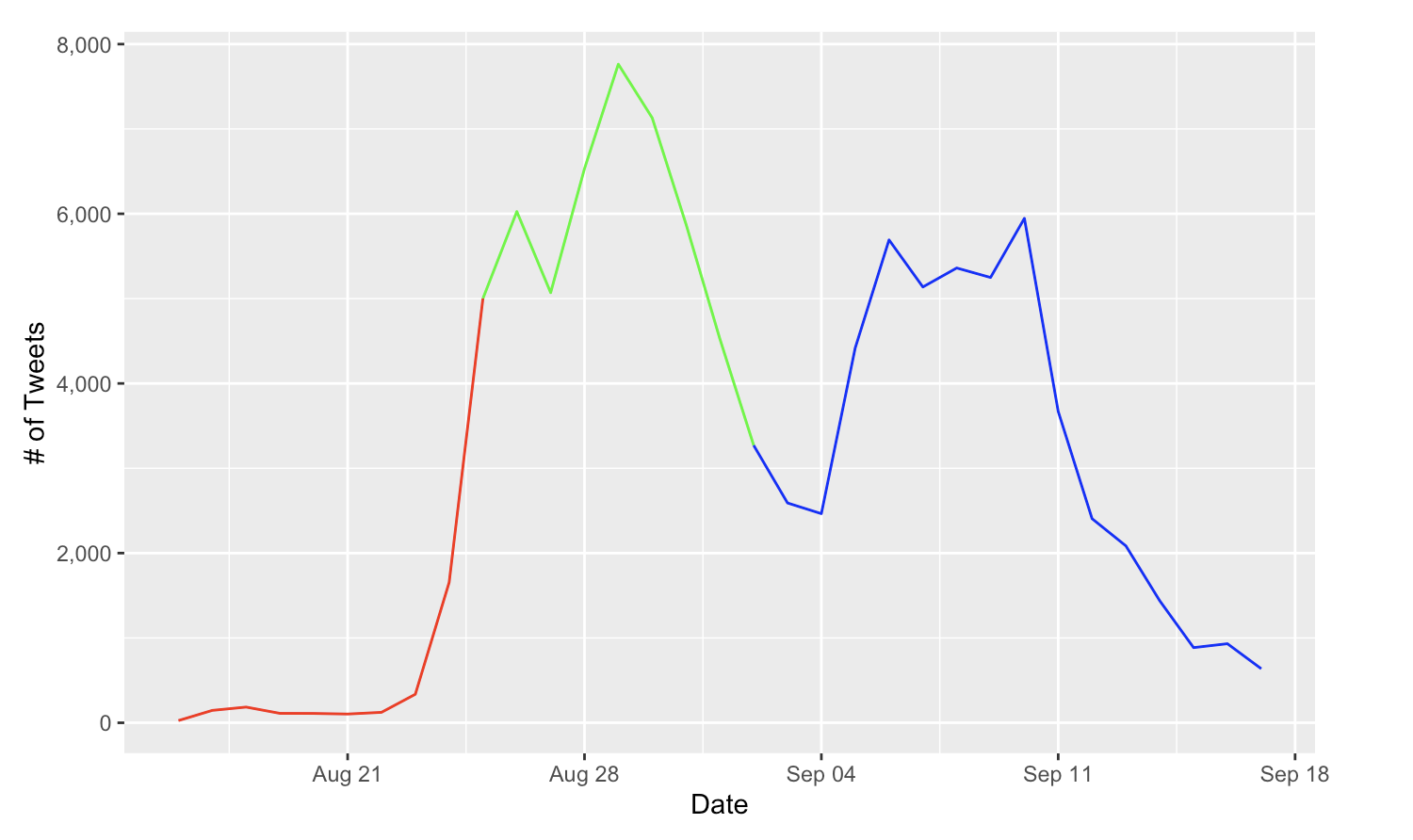

Supplement: S6 Fig — (TIF) [file pone.0316852.s007.tif]
